# Plasmodium vivax Presenting With Septic Shock and Disseminated Intravascular Coagulation (DIC): A Case Report

**DOI:** 10.7759/cureus.40042

**Published:** 2023-06-06

**Authors:** Zain Satti, Abaan Khurshid, Rahed Mohammed, Rejath Jose, Adewale Olayode

**Affiliations:** 1 School of Medicine, New York Institute of Technology College of Osteopathic Medicine, Old Westbury, USA; 2 Critical Care Medicine, Mount Sinai Queens Hospital, Queens, USA; 3 Internal Medicine, New York Institute of Technology College of Osteopathic Medicine, Old Westbury, USA

**Keywords:** disseminated intravascular coagulation (dic), septic shock [ss], infectious disease pathology, malaria treatment, plasmodium vivax malaria

## Abstract

Malaria has various causative agents that can have a spectrum of disease manifestations, some potentially fatal. Various species have been established as etiologies of malaria, though our understanding of the severity of various species is changing. We present a unique case of* Plasmodium vivax* malaria that resulted in severe disease, a magnitude rarely seen in previous literature. Our patient was a 35-year-old healthy woman who presented to the emergency department with abdominal pain, nausea, vomiting, and fever. Further workup revealed severe thrombocytopenia with prolonged prothrombin (PT) and partial thromboplastin time (PTT). An initial thick smear failed to detect any *Plasmodium *species, but a thin smear revealed *P. vivax*. The patient's hospital stay was complicated by septic shock requiring intensive care unit (ICU) admission. This unique case represents* P. vivax* as the causative agent of severe malaria even in healthy, immunocompetent patients.

## Introduction

Malaria is a potentially fatal disease transmitted from the bite of a female Anopheles spp mosquito. The Anopheles mosquito’s saliva contains Plasmodium, which is a single-celled eukaryotic organism. Despite advances in preventive and diagnostic measures, the World Health Organization (WHO) reported an estimated 247 million cases and 619 thousand deaths in 2022 alone [1]. Though there are over 100 different species of Plasmodium, the four that infect humans are *P. falciparum*, *P. vivax*, *P. ovale*, and *P. malaria* [2]. Malaria incidence occurs in close to 85 countries and territories, though 90% of the cases occur in African regions, followed by South Asia and the Eastern Mediterranean [3].

The clinical course of malaria can be varied. Following the initial infective bite, 7-30 days often pass before the onset of symptoms, which is known as the incubation period. Initial symptoms of uncomplicated disease are typically nonspecific, often including fevers, chills, malaise/fatigue, tachycardia, abdominal pain, and/or diarrhea. Fevers may initially occur at irregular intervals, but later in the course of the infection, classic febrile paroxysms may ensue (depending on the species) [4]. On physical examination, lethargy, pallor, mild jaundice, and/or a palpable spleen may be appreciated. Hematologic abnormalities may include thrombocytopenia, hyperbilirubinemia, anemia, and/or elevated hepatic aminotransferase levels [4]. 

The gold standard of diagnosis is a Giemsa-stained blood smear [5]. Thick smears are generally more sensitive and can detect parasitemia. Thin smears provide clearer visualization of parasites and are used to measure parasite density [5]. Rapid diagnostic testing is also available, though any positive rapid test requires confirmation with light microscopy.

Severe malaria is classically associated with *P. falciparum* and is a medical emergency, with mortality approaching 100% without appropriate treatment. Studies have shown that although *P. vivax* has a higher incidence, P. falciparum is associated with significantly higher rates of hospitalization and death [6]. Young and pregnant individuals are at increased risk of severe disease [6]. Characteristics of severe malaria may include severe anemia, thrombocytopenia, seizures, hypoglycemia, metabolic acidosis, and/or hemodynamic instability [4]. 

The WHO recommends combined artemisinin therapy for treating severe malaria, though the regimen varies based on regional sensitivity patterns. For patients who meet the criteria for severe malaria, the first step is to initiate intravenous (IV) or intramuscular (IM) artesunate [7]. After the third dose of artesunate, patients are generally able to transition to oral treatment in order to complete the therapy course and prevent relapse.

This case is unique as *P. vivax* rarely presents with severe malaria of this magnitude. The patient presented herein was found to have septic shock and disseminated intravascular coagulopathy (DIC), which are uncommon complications for this etiology of malaria. Additionally, an initial thin smear was sent as part of the routine hematologic analysis, but given its poor sensitivity for malaria, it failed to detect any organisms. This case displays the importance of awareness of severe *P. vivax* malaria and how timely diagnosis can lead to potentially life-saving treatment.

## Case presentation

A 35-year-old Hispanic female with no past medical history presented to the emergency department in New York City with a chief complaint of aching upper abdominal pain for four days. The abdominal pain was initially abrupt but progressively worsened, and it was associated with fatigue, fever, chills, nausea, vomiting, and “black” stools. The patient believed that her symptoms stemmed from the consumption of “wet rice” earlier in the week. In a further interview, it was revealed that about 1 month prior to the presentation, the patient traveled through the jungle as she migrated from Ecuador to the United States.

On physical examination, the patient was in acute distress and had upper abdominal tenderness to palpation, without any guarding or rebound. The patient had a fever of 101.3°F and a blood pressure of 89/49 mmHg. The blood pressure remained hypotensive despite a 4L fluid bolus, prompting critical care admission for septic shock of unknown etiology. Blood cultures, stool pathogens, viral panels, and peripheral blood smear were all ordered and the patient was initiated on empiric broad-spectrum antibiotics. CT abdomen and pelvis did not reveal any acute abdominopelvic pathology, and routine blood work revealed severe thrombocytopenia with prolonged PT and PTT, suggestive of disseminated intravascular coagulopathy (DIC) (Table 1). This was further supported by a fibrinogen level of 102 mg/dL, d-dimer of 15.89 μg/mL, and a platelet count of 22,000 per mm3 eliciting an International Society on Thrombosis and Haemostasis (ISTH) Criteria for a DIC score of five, consistent with overt DIC (Table 1). This was calculated by the findings of a platelet count <50,000 per mm3 (two points) and a severe increase in the d-dimer level (three points). Additional findings included transaminitis, elevated alkaline phosphatase, and direct hyperbilirubinemia. Gastrointestinal stool pathology, blood cultures, and various viral tests all yielded negative results. Given the patient’s thrombocytopenia, a thick peripheral blood smear was ordered but this did not reveal any abnormal pathology. Hematology was consulted, and based on their recommendation, a peripheral thin blood smear was ordered, which ultimately revealed trophozoite rings within infected reticulocytes, establishing a diagnosis of malaria. Subsequent polymerase chain reaction (PCR) revealed *P. vivax* as the causative organism of infection.

**Table 1:** Results of complete blood count, coagulation studies, and liver function tests. PT, prothrombin time; PTT, partial thromboplastin time

Test	Result	Reference values
White cell count (per mm^3^)	2.77	4,500-11,000
Hemoglobin, blood (g/dL)	15.3	13.5-17.5
Platelets (per mm^3^)	22,000	150,000-400,000
PT (s)	16.2	12.3-14.9
PTT (s)	36.4	25.4-34.9
Alkaline phosphatase (U/L)	248	25-100
Total bilirubin (mg/dL)	3.4	0.1-1.0
Aspartate aminotransferase (U/L)	264	12-38
Alanine aminotransferase (U/L)	221	10-40
Fibrinogen (mg/dL)	102	175-450
D-dimer (μg/mL)	15.89	0-0.49

Given the patient’s travel history, chloroquine-resistant *P. vivax* was assumed and the patient was started on combined artesunate therapy for severe malarial infection. Three doses of IV artesunate were followed by four doses of IV artemether/lumefantrin. Then, after glucose-6-phosphate dehydrogenase deficiency was ruled out, the patient was switched to relapse prevention therapy with oral primaquine which was completed over 15 days. Given her rapid clinical improvement, the patient was discharged from the hospital on her eighth day of admission.

## Discussion

Plasmodium vivax malaria has long been considered to cause relatively benign or mild illness [6]. However, this patient's atypical presentation along with rapid deterioration demonstrates that *P. vivax* may be more severe than appreciated. Although WHO guidelines highlight *P. falciparum* as the primary cause of severe malaria cases, this case demonstrates that *P. vivax* can also be near fatal if not managed in a timely manner. Though this presentation is seemingly rare, with one study identifying only 48 cases of severe *P. vivax* malaria between 1990 and 2012, trends seem to be changing across medical literature, with an increasing number of severe *P. vivax* cases being reported [8-12].

This patient's presentation with septic shock along with DIC is an extremely rare occurrence. Isolated case reports have reported the possibility of *P. vivax* causing cerebral malaria, renal failure, and/or respiratory distress, though few illustrate the clinical findings demonstrated by our patient [8]. A review of the literature revealed very limited case reports of isolated *P. vivax* sepsis, with most cases affecting the high-risk neonatal and elderly populations [10-12]. In our patient, an immunocompetent and otherwise healthy 35-year-old female, *P. vivax* sepsis seems to be extremely atypical. Additionally, DIC with severe thrombocytopenia, defined as 20,000-50,000 platelets per microliter, is a rare occurrence that has only been reported in 10.1% of cases [9]. Very severe thrombocytopenia, defined as <20,000 platelets per microliter, is an even rarer occurrence that has only been reported in a handful of cases [13]. Since our patient was found to have sepsis and DIC with a platelet count that bordered on very severe thrombocytopenia, this case warranted documentation in the literature.

Additionally, this case highlights the importance of utilizing both thick and thin smears to establish a malaria diagnosis. Though thick smears are 20-40 times more sensitive than thin for Plasmodium species screening, current recommendations suggest performing a 200 oil immersion at a magnification of 1000× on both thick and thin smears before reporting a negative result [4]. When both thick and thin smears are utilized, the sensitivity is 90%. This case demonstrates the importance of clinician awareness and that negative thick smear results require a thin smear follow-up.

It is important to keep in consideration the various possible manifestations of *P. vivax*, as doing so may lead to the initiation of early, potentially life-saving therapy. Additionally, early diagnosis of severe *P. vivax* malaria may help in mitigating high care costs associated with lengthy hospital and intensive care unit stays. Many studies have suggested that the paradigm of severe malaria is shifting, with one reporting 32.7% of cases in the sample population exhibiting severe characteristics when infected with *P. vivax* [8-10]. Given the higher international incidence of *P. vivax* malaria, this makes the possibility of severe infection a threat to global healthcare systems.

## Conclusions

This case of a 35-year-old female with *P. vivax* malaria leading to DIC and septic shock demonstrates the clinical importance of recognizing *P. vivax* as a possible cause of severe malaria. *P. falciparum* has long been the primary focus of severe disease, though *P. vivax* may potentially be more severe than previously recognized. The case we present here demonstrates an individual with no past medical history or risk factors, an uncommon demographic for severe *P. vivax* malaria. Her hospital stay required intensive care unit (ICU) admission for septic shock and DIC which is atypical given her age and medical history. Our case report serves to raise awareness among healthcare providers of the possible severity of *P. vivax* and the importance of timely diagnosis and treatment.
